# Leptin Promotes *cPLA_2_* Gene Expression through Activation of the MAPK/NF-κB/p300 Cascade

**DOI:** 10.3390/ijms161126045

**Published:** 2015-11-18

**Authors:** Pei-Sung Hsu, Chi-Sheng Wu, Jia-Feng Chang, Wei-Ning Lin

**Affiliations:** 1 Division of Chest Medicine, Shin Kong Wu Ho-Su Memorial Hospital, Taipei 11101, Taiwan; pack14tw@gmail.com; 2 Graduate Institute of Basic Medicine, Fu Jen Catholic University, Xinzhuang 24205, Taiwan; leo-wu@livemail.tw; 3 Department of Internal Medicine, En-Chu-Kong Hospital, Sanxia 23702, Taiwan; cjf6699@yahoo.com.tw; 4 PhD Program in Nutrition and Food Science, Fu Jen Catholic University, Xinzhuang 24205, Taiwan

**Keywords:** leptin, inflammation, MAPK, NF-κB, p300

## Abstract

Hyperplasia or hypertrophy of adipose tissues plays a crucial role in obesity, which is accompanied by the release of leptin. Recently, obesity was determined to be associated with various pulmonary diseases including asthma, acute lung injury, and chronic obstructive pulmonary disease. However, how obesity contributes to pulmonary diseases and whether leptin directly regulates lung inflammation remains unclear. We used cell and animal models to study the mechanisms of leptin mediation of pulmonary inflammation. We found that leptin activated *de novo* synthesis of cytosolic phospholipase A_2_-α (cPLA_2_-α) *in vitro* in the lung alveolar type II cells, A549, and *in vivo* in ICR mice. Upregulated cPLA_2_-α protein was attenuated by pretreatment with an OB-R blocking antibody, U0126, SB202190, SP600125, Bay11-7086, garcinol, and p300 siRNA, suggesting roles of p42/p44 MAPK, p38 MAPK, JNK1/2, NF-κB, and p300 in leptin effects. Leptin enhanced the activities of p42/p44 MAPK, p38 MAPK, JNK1/2, and p65 NF-κB in a time-dependent manner. Additional studies have suggested the participation of OB-R, p42/p44 MAPK, and JNK1/2 in leptin-increased p65 phosphorylation. Furthermore, p300 phosphorylation and histone H4 acetylation were reduced by blockage of OB-R, p42/p44 MAPK, p38 MAPK, JNK1/2, and NF-κB in leptin-stimulated cells. Similarly, blockage of the MAPKs/NF-κB/p300 cascade significantly inhibited leptin-mediated cPLA_2_-α mRNA expression. Our data as a whole showed that leptin contributed to lung cPLA_2_-α expression through OB-R-dependent activation of the MAPKs/NF-κB/p300 cascade.

## 1. Introduction

Obesity, a worldwide epidemic, is a type of health impairment resulting from aberrant or excessive adipose accumulation [1]. Recently, obesity has been found to relate to various pulmonary diseases, including obstructive sleep apnea, asthma, acute lung injury (ALI), and chronic obstructive pulmonary disease (COPD) [2,3,4,5]. It is known that an increase in body mass index (BMI, kg/m^2^) is associated with an elevation in the incidence of asthma [6] and that people with obesity who undergo surgery to remove approximately 30% of their weight are 2 to 7.5 times less likely to have obstructive sleep apnea symptoms two years post-surgery [7]. Moreover, epidemiological studies have revealed that obesity is a crucial predisposing factor in the development of ALI [5]. However, how obesity contributes to the occurrence of pulmonary diseases is largely unknown. It is known, however, that a pulmonary inflammatory response occurs during recovery from exogenous infection or injury of the lungs and can result in lasting damage if not properly resolved. Inflammation-related proteins such as cytosolic phospholipase A_2_-α (cPLA_2_-α) are upregulated in response to the stimulation of exogenous stimuli or cytokines in the lung system [8,9]. It was found that the expression of cPLA_2_-α exacerbated *Pseudomonas aeruginosa*-induced lung injury and mice lethality [10]. In addition, laboratory cPLA_2_-null mice showed less pulmonary edema, neutrophil sequestration, and deterioration of gas exchange compared with mice administered lipopolysaccharide or zymosan or with those suffering from acid aspiration-induced acute respiratory distress syndrome [11,12]. This suggested that cPLA_2_-α contributed to the severity of lung diseases and that the inhibition of a cPLA_2_-α-mediated pathway may provide a therapeutic approach against lung pathologies. However, whether obesity contributes to lung pathologies by upregulating the expression of cPLA_2_-α and its related mechanisms remains unknown. Thus, the mechanisms underlying obesity-induced cPLA_2_-α expression were investigated in human alveolar type II A549 cells.

Adipose tissue is composed of adipocytes and a vascular-stromal fraction, which contains macrophages, fibroblasts, endothelial cells, and preadipocytes [13]. In addition to the regulation of whole-body fatty acid homeostasis, adipose tissue functions as a complex endocrine organ, secreting adipokines [14]. Leptin, a 16-kDa adipocyte-derived adipokine, is the product of the obesity (Ob) gene. Structurally similar to IL-6, IL-11, and IL-12, leptin belongs to the long-chain helical cytokine family and functions as an immune modulator [15]. Leptin activates macrophages/monocytes and natural killer cells, and regulates the proliferation, phagocytosis, chemotaxis, and oxygen radical release of neutrophils [16]. Leptin also promotes naïve T cell proliferation and IL-2 secretion, the memory T cells proliferation and differentiation toward T helper 1 cell immune responses, and the suppression of the CD4^+^CD25^+^ regulatory T cell proliferation [16,17]. Apoptosis of T cells are prevented by leptin through the upregulation of the anti-apoptotic protein bcl-xL [16]. Although experiments have indicated the roles of leptin in mediating immune responses, fewer studies examine their roles in pulmonary inflammation. Thus, obesity and pulmonary inflammation were correlated by investigating leptin effects on regulating cPLA_2_-α expressions.

Once released, leptin affects cells by binding to the leptin receptor OB-R, the product of the diabetes (db) gene. OB-R belongs to the class I cytokine receptor family with no intrinsic tyrosine kinase activity and is activated by the formation of homo- or heterodimers [18]. Six OB-R isoforms containing identical N-terminal binding domains but different lengths of cytoplasmic domains were identified: OB-Ra, OB-Rb, OB-Rc, OB-Rd, OB-Rf, and OB-Re [19,20]. However, with its approximately 300 intracellular residues, OB-Rb is the only one isoform to transduce downstream signaling [21]. After binding to OB-R, leptin activates the mitogen-activated protein kinase (MAPK) cascade by recruiting SH2-containing tyrosine phosphatase-2 (SHP2; PTPN11) [22,23,24]. Mammalian MAPKs are composed of p42/p44 MAPK, p38 MAPK, and JNK1/2. A previous study indicated that p42/p44 MAPK and JNK1/2 participated in leptin-stimulated MMP-1 expression, but p42/p44 MAPK and p38 MAPK played roles in MMP13 expression in leptin-stimulated rat nucleus pulposus cells [25]. However, whether leptin-contributed expression of cPLA_2_-α in the lung occurred through the activation of MAPKs remained unknown. In addition, activation of nuclear factor-κB (NF-κB) and p300 were demonstrated to drive gene activation of cPLA_2_ [26,27]. It was shown that the increase of NF-κB activity was unrelated to the phosphorylation of MAPKs under the stimulation of TNF-α or IL-1β, but lipopolysaccharide was shown to phosphorylate NF-κB through MAPKs [28,29,30]. Whether NF-κB and p300 added to leptin-contributed cPLA_2_-α expression and its related mechanisms remain unclear.

We hypothesized that leptin contributes to pulmonary pathologies by upregulating cPLA_2_-α expression. The results revealed that leptin increased *de novo* expression of cPLA_2_-α in human alveolar type II A549 cells and in ICR mice. Pretreatment with MAPKs, NF-κB or p300 inhibitors suggested the participation of the MAPKs, NF-κB and p300 signal components in the gene activation process, with the attenuation of the leptin-induced expression of cPLA_2_-α yielding a similar indication. Leptin also stimulated the phosphorylation of MAPKs, NF-κB, and p300. However, leptin-induced phosphorylation of NF-κB was attenuated by inhibitors of p42/p44 MAPK and JNK1/2 but not p38 MAPK. Similarly, phosphorylation of p300 resulted in acetylation of histone H4 but was attenuated by MAPKs and NF-κB inhibitors. In conclusion, we showed that leptin mediated cPLA_2_-α expression in A549 cells through p42/p44 MAPK and JNK1/2-dependent NF-κB and p300 activation.

## 2. Results

### 2.1. In Vitro and in Vivo Expression of cPLA_2_-*α* in Response to Leptin Stimulation

**Figure 1:**
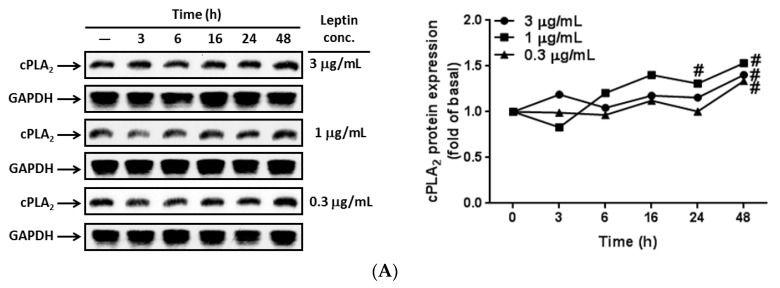

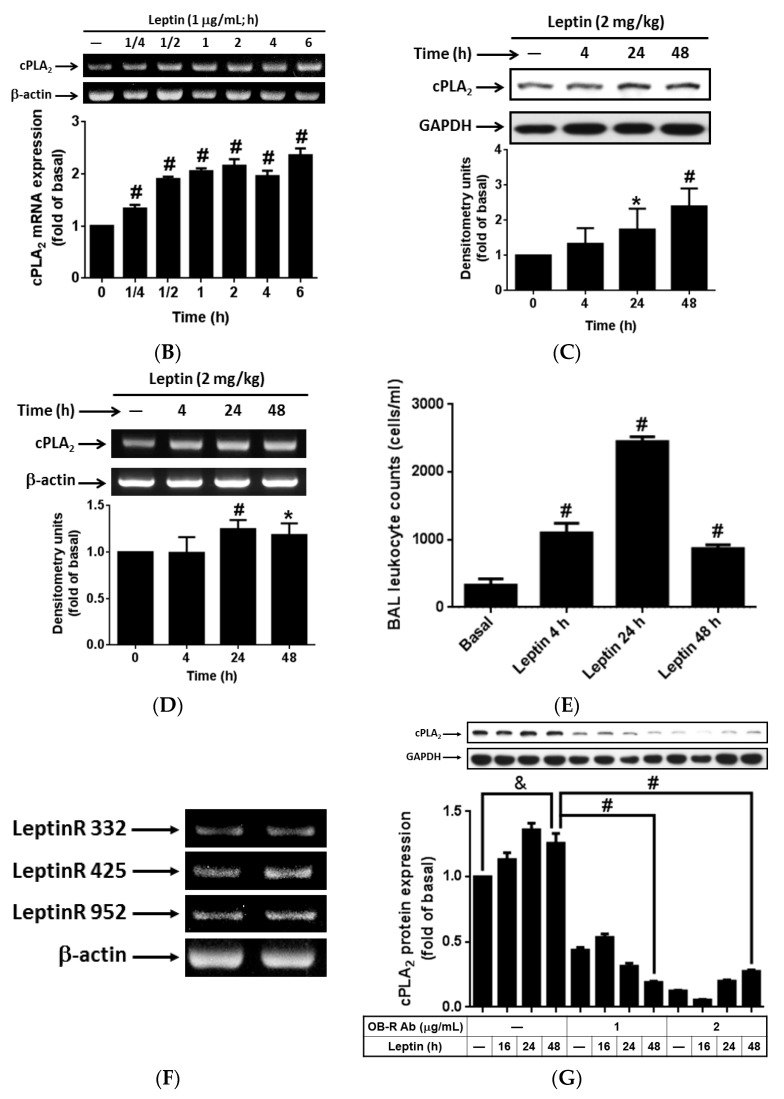
Leptin-stimulated gene expression of *cPLA_2_*-α. (**A**,**B**) A549 cells were incubated with various concentrations or 1 µg/mL of leptin for the indicated time intervals. Expression of cPLA_2_-α was determined using (**A**) Western blot and (**B**) RT-PCR; (**C**–**E**) The ICR mice were treated with leptin (2 mg/kg) for the indicated time points. After the mice were sacrificed, lung tissues and bronchoalveolar lavage (BAL) fluid were extracted as described in the Experimental section; (**C**) Western blot was used to determine the protein expression; (**D**) RT-PCR was used to determine the mRNA expression and (**E**) leukocytes in BAL fluid were counted using a hematology analyzer. Data are expressed as mean ± SEM of 5 independent experiments (*n* = 5). # *p <* 0.01 or * *p <* 0.05 as compared with the cells exposed to the vehicle alone; (**F**) Expression of leptin receptor isotypes were examined using RT-PCR; (**G**) A549 cells were pretreated with 1 or 2 µg/mL of the OB-R antibody for 1 h and then incubated with 1 µg/mL of leptin for the indicated time intervals. The expression of cPLA_2_-α protein was determined using Western blot. The data are expressed as mean ± SEM of five independent experiments (*n* = 5). & *p* < 0.05 as compared with the cells exposed to vehicle alone; # *p* < 0.01 as compared with the cells exposed to leptin.

To examine the effects of leptin on the lungs, A549 cells were growth arrested and incubated using different concentrations of leptin for the various time intervals. At the end of incubation, the cells were lysed and their protein was extracted for detecting cPLA_2_-α expression by using Western blot. Proteins were loaded into a 10% concentration SDS-PAGE and probed with an anti-cPLA_2_-α antibody. The same membranes were stripped and reprobed with the anti-GAPDH antibody as internal controls. The expression of cPLA_2_-α was upregulated in response to leptin stimulation in a time-dependent manner with maximum responses occurring at 48 h of leptin stimulation (Figure 1A). We also observed that 1 µg/mL of leptin showed higher levels of cPLA_2_-α expression than the other two leptin concentrations (Figure 1A). To investigate mRNA expression, serum-starved A549 cells were treated with 1 µg/mL of leptin for the indicated time intervals (Figure 1B). Subsequently, mRNA was extracted as a template of cDNA, and the expression of cPLA_2_-α mRNA was detected using RT-PCR. We determined that leptin stimulated expression of cPLA_2_-α mRNA, time-dependently, with maximum responses occurring at 6 h (Figure 1B). To determine whether leptin contributed to *cPLA_2_-*α gene expression *in vivo*, ICR mice were anesthetized and given an intratracheal injection of 2 mg/kg of leptin into their lungs. After 4, 24, or 48 h, the mice were anesthetized again and bronchoalveolar lavage (BAL) was performed to analyze changes in leukocyte levels in the lungs. After the mice were sacrificed, mRNA and protein were extracted from isolated lung tissues. The protein and mRNA expression of cPLA_2_-α was investigated using Western blot and RT-PCR, respectively. Both the protein and mRNA expression of cPLA_2_-α were upregulated after leptin treatment (Figure 1C,D). Moreover, there was increased leukocyte accumulation in the leptin-stimulated lungs (Figure 1E). Leptin-stimulated leukocyte accumulation peaked at 24 h; however, even after decreasing, it still remained higher than basal levels. We determined that leptin-stimulated a cPLA_2_-α increase *in vitro* in A549 cells and *in vivo* in ICR mice. Moreover, we showed that A549 cells expressed mRNA of OB-R (Figure 1F,G). To determine whether leptin contributed to cPLA2-α expression through its receptors, cells were pretreated with 1 or 2 µg/mL of OB-R for 1 h and then stimulated with 1 µg/mL of leptin for 0, 16, 24, or 48 h. Data from Western blots revealed that leptin-upregulated cPLA2-α expression was attenuated by an OB-R blocking antibody (Figure 1G). These data suggested that leptin increased *cPLA_2_*-α gene expression through OB-R in the A549 cells.

### 2.2. Phosphorylated p42/p44 MAPK Contributed to Leptin-Stimulated cPLA_2_-*α* Gene Expression

**Figure 2:**
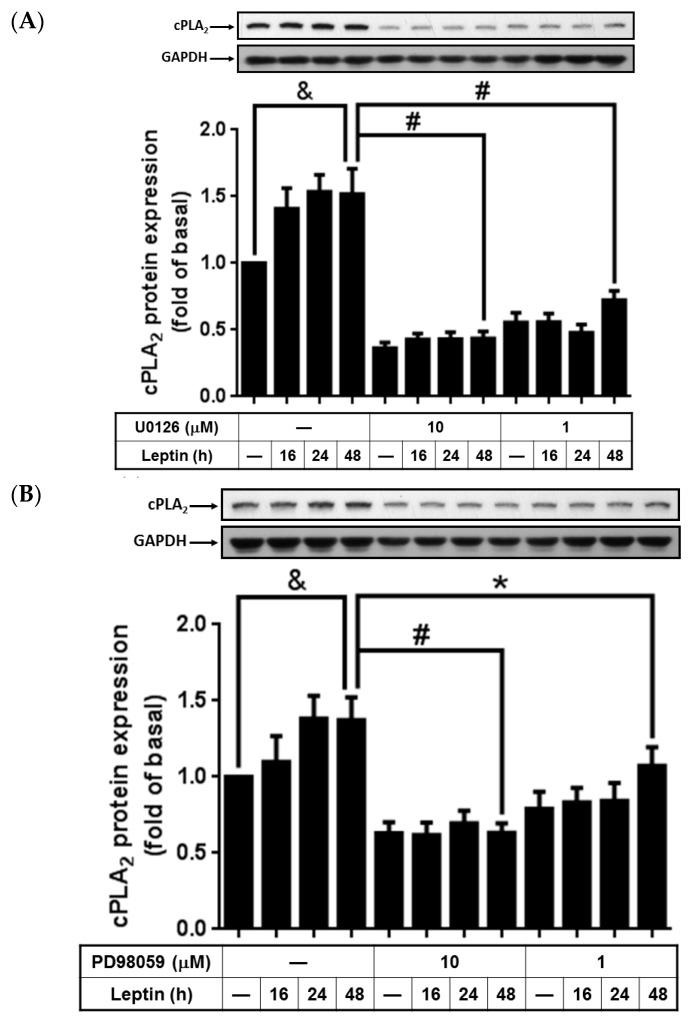

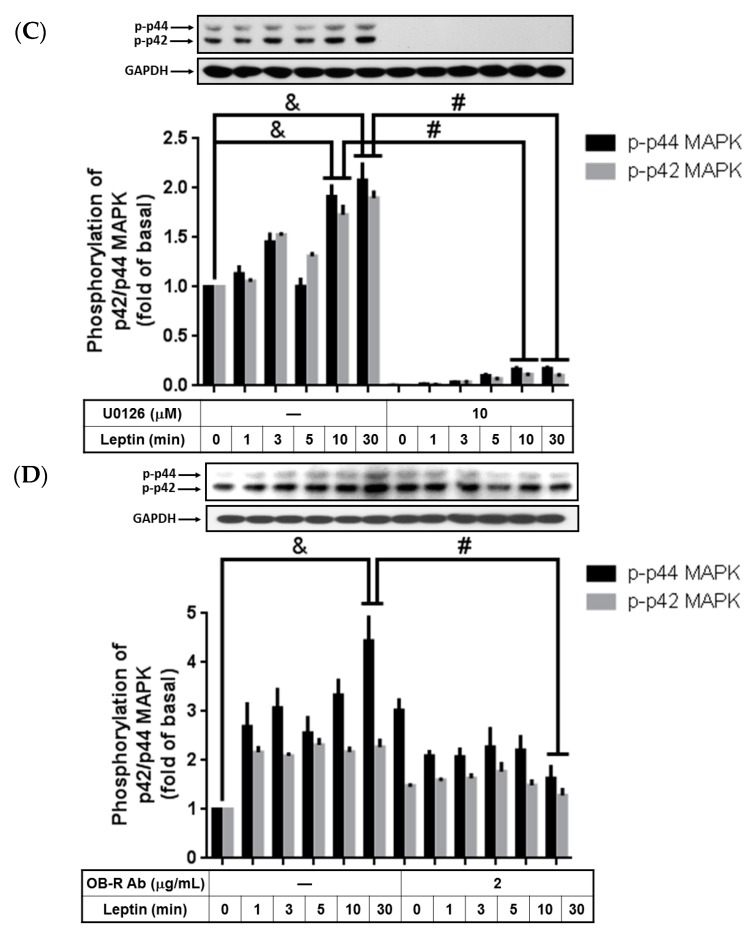
Pretreatment with PD98059 and U0126 on leptin-stimulated A549 cells. Serum-starved A549 cells were pretreated with different concentrations of U0126, PD98059, or the OB-R antibody for 1 h and then stimulated by 1 µg/mL of leptin for the indicated time intervals. At the end of incubation, the cells were harvested and cell lysates were extracted. (**A**,**B**) Expression of cPLA_2_-α and (**C**,**D**) phosphorylation of p42/p44 MAPK were detected using Western blot with anti-cPLA_2_ or anti-phospho p42/p44 MAPK antibodies. Cell membranes were stripped and reprobed with the anti-GAPDH antibody as internal controls. The data are expressed as mean ± SEM of five independent experiments (*n* = 5). & *p* < 0.05 as compared with the cells exposed to the vehicle alone; # *p* < 0.01 or * *p* < 0.05 as compared with the cells exposed to leptin.

It has been determined that the *cPLA_2_*-α gene is regulated by p42/p44 MAPK in response to stimulation by TNF-α and IL-1β in tracheal smooth-muscle cells or rheumatoid arthritis fibroblasts [9,31]. To determine whether activation of p42/p44 MAPK would also contribute to leptin-induced cPLA_2_-α expression, A549 cells were pretreated with various concentrations of MEK1/2 inhibitors, PD98059, or U0126 for 1 h and then incubated with 1 µg/mL of leptin for 0, 16, 24, or 48 h. PD98059 and U0126 pretreatment significantly blocked leptin-stimulated cPLA_2_-α expression (Figure 2A,B). To determine the contributing effects of leptin on p42/p44 MAPK activity, serum-starved A549 cells were either pretreated with or without the OB-R blocking antibody (2 µg/mL) or U0126 (10 µM), and then incubated with 1 µg/mL of leptin for the indicated time intervals. In the non-pretreated cells, leptin stimulated phosphorylation of p42/p44 MAPK in a time-dependent pattern, with a maximum response occurring at 30 min after leptin incubation (Figure 2C,D). Pretreatment with either the OB-R antibody or U0126 abolished leptin-increased p42/p44 MAPK phosphorylation, suggesting that leptin contributed to p42/p44 MAPK activation through OB-R and MEK1/2 (Figure 2C,D). Overall, these data revealed that leptin upregulated cPLA_2_-α protein expression through OB-R.

### 2.3. Activation of p38 MAPK Involved in Leptin-Stimulated cPLA_2_-α Expression

**Figure 3:**
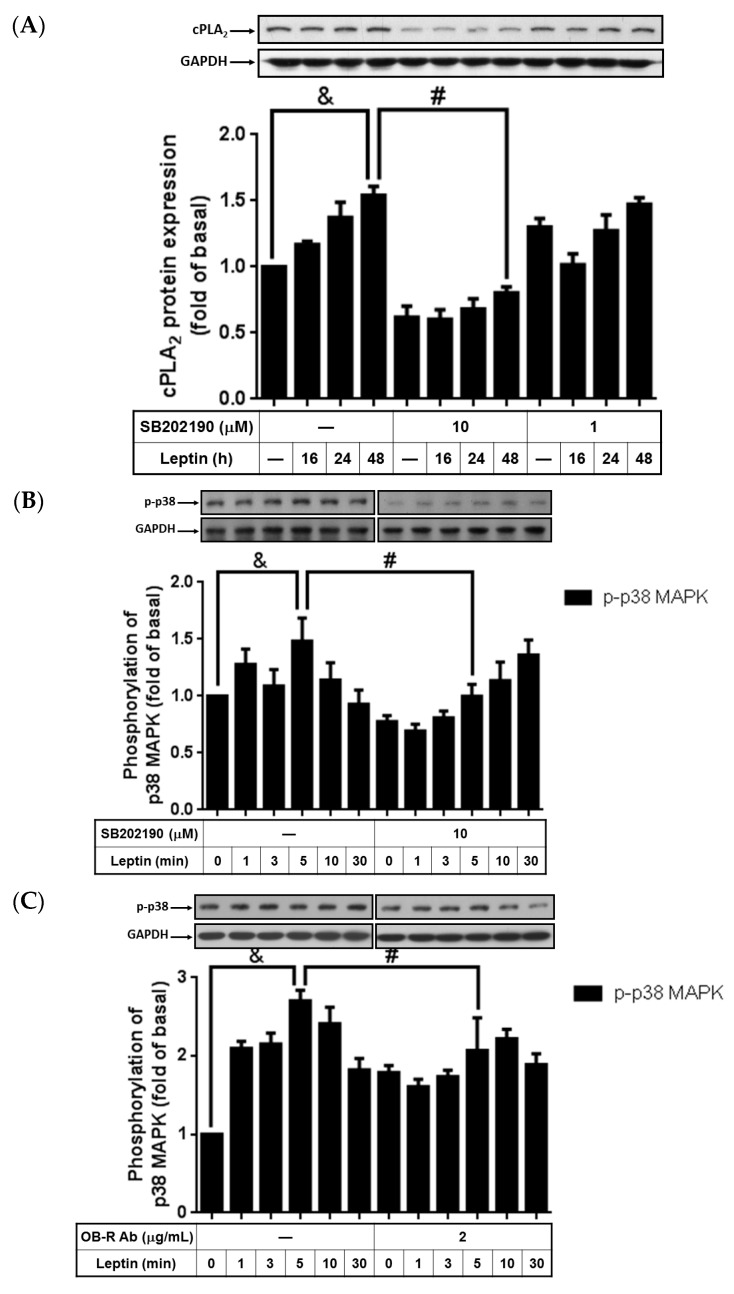
SB202190 attenuation of cPLA_2_-α expression and p38 MAPK phosphorylation. Serum-starved A549 cells were pretreated with different concentrations of SB202190 or the OB-R antibody for 1 h and then stimulated by 1 µg/mL of leptin for the indicated time intervals. At the end of incubation, the cells were harvested and cell lysates were extracted. (**A**) Expression of cPLA_2_-α and (**B**,**C**) phosphorylation of p38 MAPK were detected using Western blot with specific antibodies. Cell membranes were stripped and reprobed with the anti-GAPDH antibody as internal controls. The data are expressed as mean ± SEM of five independent experiments (*n* = 5). & *p* < 0.05 as compared with the cells exposed to the vehicle alone; # *p* < 0.01 as compared with the cells exposed to leptin.

Evidence indicated that leptin changed cell phenotypes or downregulated aggrecanase expression through the p38 MAPK pathway [32,33]. To study whether p38 MAPK participated in leptin-regulated cPLA_2_-α expression, cells were incubated with 10 or 1 µM p38 MAPK inhibitor (SB202190) for 1 h, and treated with leptin (1 µg/mL) for another 0, 16, 24, or 48 h. After incubation, the cells were harvested and proteins were subjected to a 10% concentration SDS-PAGE to analyze the cPLA-_2_α protein expression. We found that leptin-induced cPLA_2_-α expression was attenuated by pretreatment with 10 µM SB202190 (Figure 3A). This suggested that p38 MAPK may participate in leptin-induced cPLA_2_-α expression. To ascertain the role of p38 MAPK in leptin effects, cells were treated with 1 µg/mL of leptin for 0, 1, 3, 5, 10, or 30 min. At the end of leptin stimulation, the cells were lysed and phosphorylation of p38 MAPK was detected using Western blot. We found that leptin increased phosphorylation of p38 MAPK, peaking at 5 min of leptin incubation (Figure 3B). Moreover, pretreatment with the OB-R blocking antibody or SB202190 significantly delayed leptin-stimulated p38 MAPK phosphorylation, suggesting that leptin contributed to p38 MAPK activation through OB-R (Figure 3C). In summary, these data revealed that leptin regulated cPLA_2_-α protein expression through OB-R-dependent activation of p38 MAPK.

### 2.4. Participation of Activated JNK1/2 in Leptin-Upregulated cPLA_2_-α Expression

**Figure 4:**
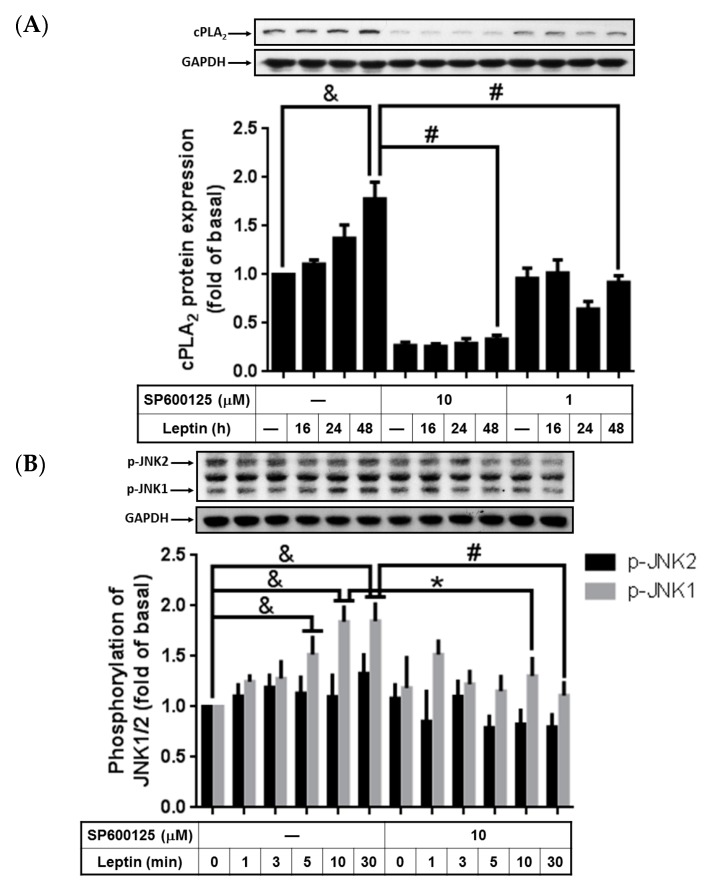
Leptin-mediated cPLA_2_-α expression and JNK1/2 phosphorylation were inhibited by SP600125. Serum-starved A549 cells were pretreated with different concentrations of SP600125 for 1 h and then stimulated by 1 µg/mL of leptin for the indicated time intervals. At the end of incubation, the cells were harvested and cell lysates were extracted. (**A**) Expression levels of cPLA_2_-α and (**B**) phosphorylation of JNK1/2 were detected using Western blot with specific antibodies. Cell membranes were stripped and reprobed with the anti-GAPDH antibody as internal controls. The data are expressed as mean ± SEM of five independent experiments (*n* = 5); & *p* < 0.05 as compared with the cells exposed to the vehicle alone; # *p* < 0.01 or * *p* < 0.05 as compared with the cells exposed to leptin.

Activation of JNK1/2 has been suggested to contribute to *cPLA_2_*-α gene activation [8,12,34]. However, whether leptin contributes to *cPLA_2_*-α gene expression through activated JNK1/2 remains unknown. To study whether activated JNK1/2 plays a role in leptin-upregulated cPLA_2_-α expression, SP600125, a specific inhibitor of JNK1/2, was used. Serum-starved A549 cells were incubated with various concentrations of SP600125 for 1 h and then stimulated with leptin (1 µg/mL) for the indicated time intervals (Figure 4A). The leptin-stimulated cPLA_2_-α expression was significantly abolished by SP600125, implying that JNK1/2 may be involved in cPLA_2_-α expression within leptin-stimulated A549 cells (Figure 4A). To evaluate whether leptin contributes to the activation of JNK1/2, A549 cells were stimulated by 1 µg/mL of leptin for the indicated time intervals (Figure 4B). Leptin enhanced time-dependently phosphorylation of JNK1, peaking at 10 min and sustaining phosphorylation for up to 30 min of leptin treatment (Figure 4B). Pretreatment with the OB-R blocking antibody or SP600125 significantly attenuated leptin-stimulated JNK1 phosphorylation. These data suggested that leptin-stimulated cPLA_2_-α expression through OB-R-dependent activation of JNK.

### 2.5. Leptin Stimulated cPLA_2_-*α* Expression via Activation of NF-κB

**Figure 5:**
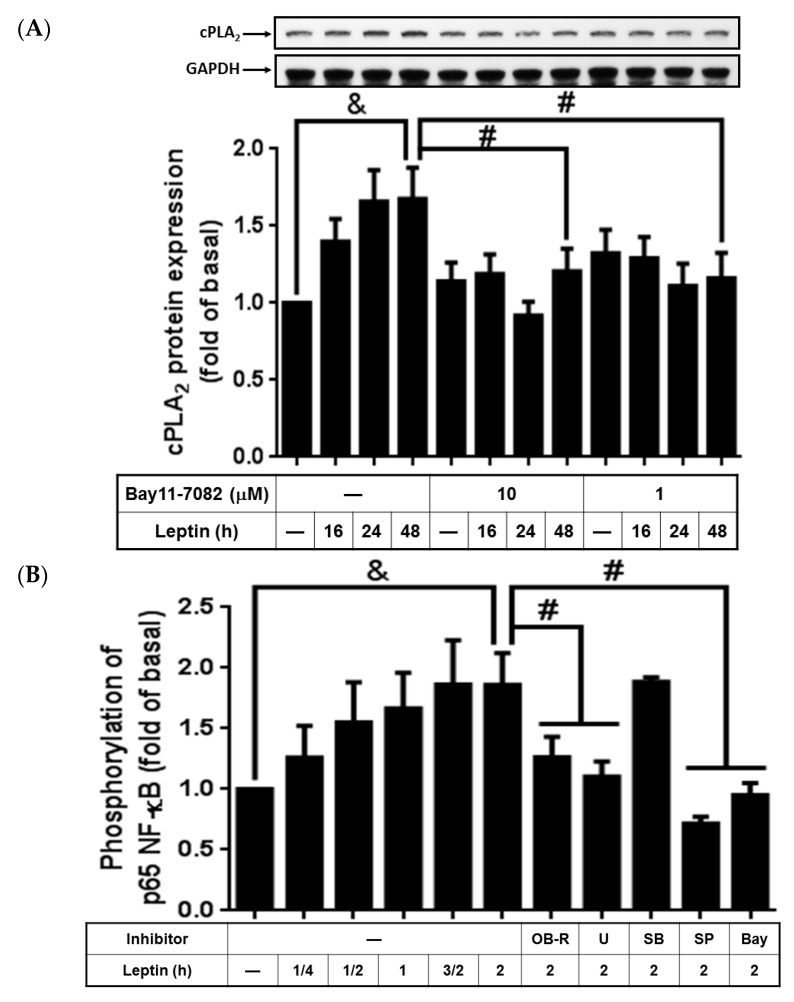
Effects of Bay11-7082 on leptin-regulated cPLA_2_-α expression and p65 phosphorylation. Serum-starved A549 cells were pretreated with different concentrations of Bay11-7082, OB-R (2 µg/mL), U0126 (10 µM), SB202190 (10 µM), SP600125 (10 µM), or Bay11-7082 (10 µM) for 1 h. The cells were then stimulated by 1 µg/mL of leptin for the indicated time intervals. At the end of incubation, the cells were harvested and cell lysates were extracted. (**A**) Expression of cPLA_2_-α and (**B**) Ser276 phosphorylation of p65 were detected using Western blot with specific antibodies. The cell membranes were stripped and reprobed with the anti-GAPDH antibody as internal controls. The data are expressed as mean ± SEM of five independent experiments (*n* = 5). & *p* < 0.05 as compared with the cells exposed to the vehicle alone; # *p* < 0.01 as compared with the cells exposed to leptin.

It was shown that leptin deficiency leads to decreased stimulation of NF-κB [35]. To elucidate if NF-κB participated in leptin-stimulated cPLA_2_-α expression, Bay11-7082, an inhibitor of IκB phosphorylation, was used. We found that leptin upregulated expression of cPLA_2_-α, which was significantly attenuated by Bay11-7082 (Figure 5A). Leptin-stimulated phosphorylation of p65, one of the NF-κB subunits, but this was abrogated by pretreatment with Bay11-7082 (Figure 5B). Moreover, pretreatment with the OB-R blocking antibody, U0126, SB202190, or SP600125 significantly attenuated NF-κB p65 subunit phosphorylation in leptin-treated A549 cells (Figure 5B). This suggested that leptin upregulated NF-κB phosphorylation through OB-R-dependent activation of MAPKs. Taken together, the findings of the study indicated that leptin increased cPLA_2_-α expression through the OB-R/MAPKs/NF-κB cascade.

### 2.6. Activated p300 Contributes to Leptin-Increased cPLA_2_-α Expression

**Figure 6:**
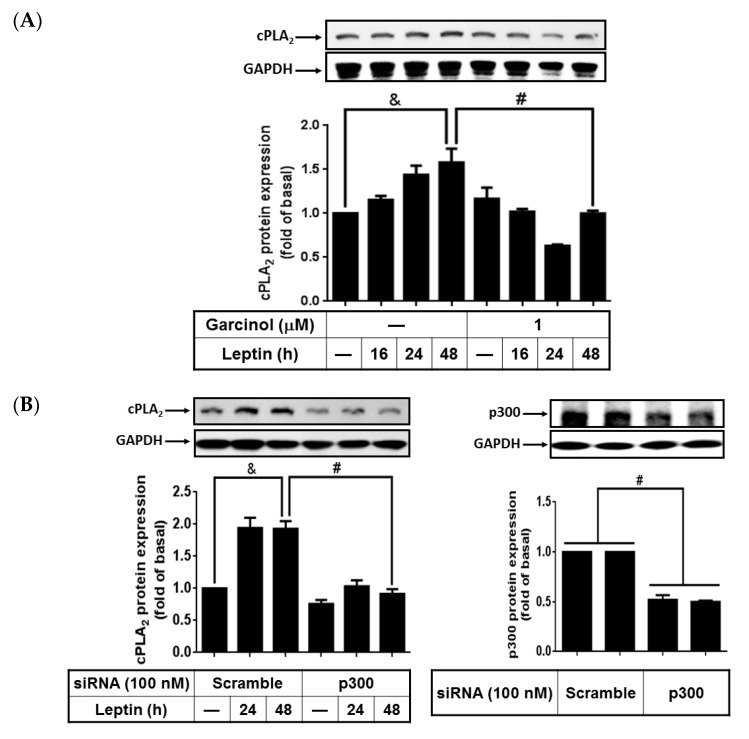

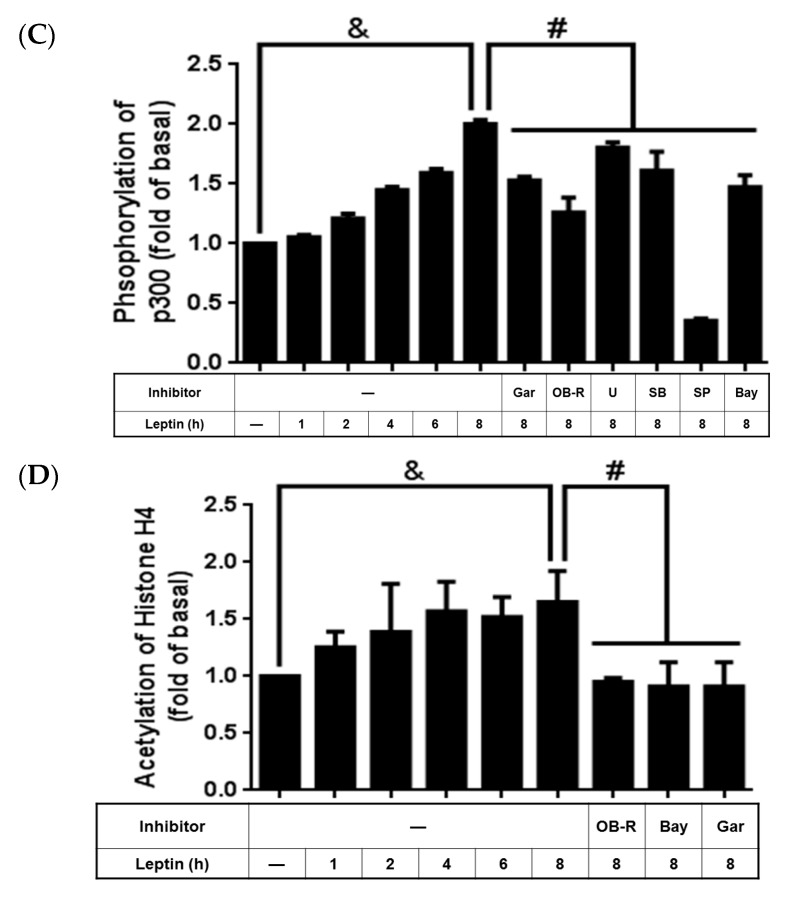
Effects of garcinol and p300 siRNA on leptin-stimulated A549 cells. Serum-starved A549 cells were pretreated with the OB-R antibody (2 µg/mL), U0126 (10 µM), SB202190 (10 µM), SP600125 (10 µM), Bay11-7082 (10 µM), or garcinol (1 µM) for 1 h. Other cells were transfected with 100 nM p300 siRNA or scrambled siRNA as described in the Experimental section. The cells were then incubated with 1 µg/mL of leptin for the indicated time intervals. At the end of the incubation, the cells were harvested and cell lysates were extracted. (**A**,**B**) Expression of cPLA_2_-α and p300; (**C**) phosphorylation of p65; and (**D**) acetylation of histone H4 were detected using Western blot with specific antibodies. Cell membranes were stripped and reprobed with the anti-GAPDH antibody as internal controls. The data are expressed as mean ± SEM of 5 independent experiments (*n* = 5). & *p* < 0.05 as compared with the cells exposed to the vehicle alone; # *p* < 0.01 as compared with the cells exposed to the leptin or scrambled siRNA transfected groups.

Previous studies showed that p300, a histone acetyltransferase, playes a role in regulating *cPLA_2_*-α gene expression [26,27]. However, whether leptin enhanced expression of cPLA_2_-α proteins by regulating p300 activity remained largely unclear. To demonstrate the role of p300 in regulating cPLA_2_-α expression in leptin-stimulated A549 cells, serum-starved cells were pretreated with 1 µM garcinol for 1 h then stimulated with 1 µg/mL of leptin for 0, 16, 24, or 48 h. Cell lysates were extracted and subjected to a 10% concentration SDS-PAGE. Pretreatment with garcinol significantly attenuated leptin-induced cPLA_2_-α expression in the A549 cells (Figure 6A). To confirm the role of p300 in regulating cPLA_2_-α expression in leptin-treated cells, p300 siRNA was used. We transfected the A549 cells with 100 nM scrambled siRNA or p300 siRNA for 24 h; they were then serum-starved for 72 h for the knockdown of p300 protein expression and were subsequently treated with 1 µg/mL of leptin for 0, 24 or 48 h. The cells were harvested and protein expression detected using Western blot. We observed that p300 siRNA significantly knocked down the protein expression of p300 and abrogated leptin-stimulated cPLA_2_-α expression in the A549 cells (Figure 6B). To determine whether leptin stimulated the activation of p300, phosphorylation of p300 in leptin-stimulated A549 cells was detected using Western blot. We observed that leptin stimulated phosphorylation of p300 in a time-dependent manner with the maximum response occurring at 8 h after leptin stimulation (Figure 6C). Pretreatment with the OB-R blocking antibody, U0126, SB202190, SP600125, Bay11-7082, or garcinol significantly reduced p300 phosphorylation in leptin-treated cells (Figure 6C). Moreover, leptin increased the acetylation of histone H4, which was attenuated by pretreatment with the OB-R antibody, Bay11-7082, and garcinol (Figure 6D). This suggested that leptin increased activation of both p300 and acetylation of histone H4 through OB-R-dependent activation of the MAPKs/NF-κB pathway. Collectively, these data indicated that leptin-stimulated cPLA_2_-α expression was regulated through activation of the OB-R/MAPKs/NF-κB/p300 pathway in A549 cells.

### 2.7. Leptin Induced cPLA_2_-*α* mRNA Expression via Ob-R/MAPK/NF-κB/p300 Cascade

**Figure 7:**
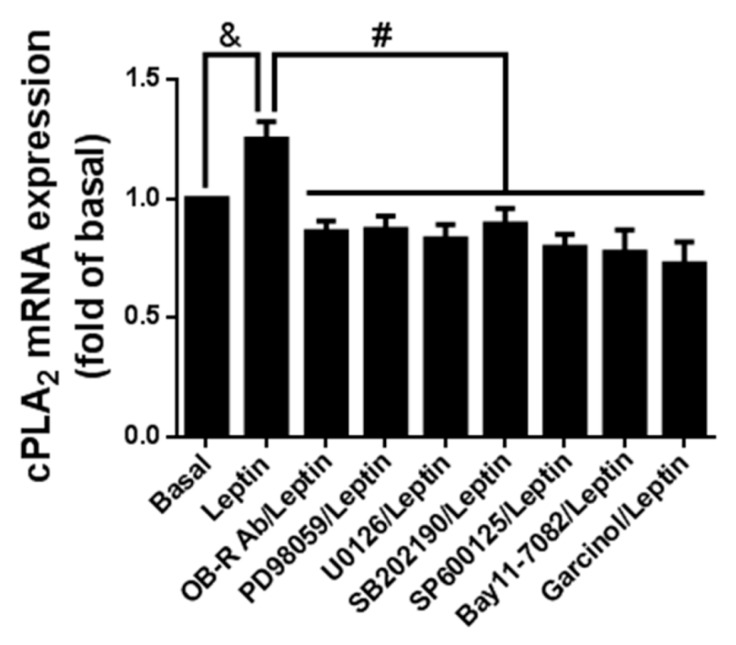
Effects of various inhibitors on leptin-stimulated cPLA_2_-α mRNA expression. Serum-starved A549 cells were pretreated with OB-R (2 µg/mL), PD98059 (10 µM), U0126 (10 µM), SB202190 (10 µM), SP600125 (10 µM), Bay11-7082 (10 µM), or garcinol (1 µM) for 1 h. The cells were then incubated with 1 µg/mL of leptin for 6 h. At the end of incubation, mRNA were extracted and used as templates of cDNA. Expression of cPLA_2_-α mRNA was detected using RT-PCR. Data are expressed as mean ± SEM of five independent experiments (*n* = 5). & *p* < 0.05 as compared with the cells exposed to the vehicle alone; # *p* < 0.01 as compared with the cells exposed to leptin.

To determine whether leptin contributed to cPLA_2_-α mRNA expression through the OB-R/MAPKs/NF-κB/p300 pathway, serum-starved cells were pretreated with the OB-R blocking antibody, U0126, SB202190, SP600125, Bay11-7082, or garcinol for 1 h and then incubated with 1 µg/mL of leptin for another 6 h. At the end of treatment, the cells were harvested and mRNA was extracted. RT-PCR was used to analyze the expression of cPLA_2_-α mRNA. Leptin stimulated cPLA_2_-α mRNA expression but was significantly attenuated by pretreatment with the OB-R blocking antibody, U0126, SB202190, SP600125, Bay11-7082, or garcinol (Figure 7). These data suggest that cPLA_2_-α expression was mediated by the OB-R/MAPKs/NF-κB/p300 signaling cascades in leptin-stimulated A549 cells.

## 3. Discussion

Obesity occurs through hyperplasia or hypertrophy of adipose tissues, a type of endocrine organ. Leptin, encoded by the *Ob* gene, is released when a mass of adipose tissue is enlarged. In addition to cardiovascular and metabolic diseases, obesity increases the risks of acute and chronic pulmonary diseases such as asthma, ALI, and COPD [2,3,4,5]. However, how obesity contributes to pulmonary diseases remained largely unknown. This study focused on the mechanisms of leptin that concern pulmonary pathologies and their relation to body fat. We found that leptin activated *de novo* synthesis of cPLA_2_-α *in vitro* in lung alveolar type II A549 cells. The increase of cPLA_2_-α protein and mRNA was also observed in the lung tissue of leptin-treated mice. Moreover, we found that upregulated expression of the *cPLA_2_*-α gene was attenuated by pretreatment with the OB-R blocking antibody, U0126, SB202190, SP600125, Bay11-7086, garcinol, and p300 siRNA. Leptin stimulated activation of p42/p44 MAPK, p38 MAPK, JNK1/2, and NF-κB in time-dependent manners. In addition, leptin-mediated phosphorylation of NF-κB was attenuated by pretreatment with the OB-R blocking antibody, U0126, SP600125, and Bay11-7086, suggesting that leptin regulated NF-κB activity through receptor-dependent activation of p42/p44 MAPK and JNK1/2. Similarly, leptin contributed to p300 activity and histone H4 acetylation, both of which were reversed by blockage of OB-R and inactivation of NF-κB and p300. Moreover, attenuation of MAPKs, NF-κB, and p300 significantly inhibited leptin-mediated cPLA_2_-α mRNA expression. In summation, we showed that leptin contributed to lung cPLA_2_-α expression through OB-R-dependent activation of the MAPK/NF-κB/p300 cascade.

It is becoming increasingly apparent that leptin, as an adipokine, plays a role in regulating lung pathologies. Leptin has been determined to contribute to the occurrence of pulmonary arterial hypertension; blockage of leptin signaling reduces the severity of pulmonary hypertension [36]. Plasma leptin concentrations and the leptin/adiponectin ratio exhibited a significantly inversed correlation with initial FEV1 in emphysema patients [37]. Leptin was detected in the alveolar lining fluid of healthy humans [38] and was induced in injured human and murine lungs [39]. In rat alveolar macrophages and murine peritoneal macrophages models, leptin increased phospholipase activity and enhanced the group IVC iPLA2 (cPLA2γ) protein expression, resulting in upregulated alveolar macrophage leukotriene synthesis [40]. Leptin contributed to the development of pulmonary neutrophilia in infections and ALI by inhibiting the apoptosis of airspace neutrophil [39]. However, the influence of leptin on pulmonary resident cells and how leptin contributes to inflammation in the resident lung cells remain unclear. We report that leptin enhanced the mRNA and protein expression of cPLA_2_-α, which functions as an inflammation marker, *in vitro* in lung alveolar cells and *in vivo* in lung tissues. This conclusion is similar to previous findings that leptin induced gene expression of intracellular adhesion molecular-1, CCL11, VEGF, G-CSF, IL-6, and cell migration on the human airway epithelial cell line [41]. This is also similar to the conclusion that leptin triggers A549 cell proliferation through blockage of endoplasmic reticulum stress-related apoptosis [39]. In this study, increased cPLA_2_-α expression was showed after stimulation of 1 µg/mL leptin for 24 to 48 h. The leptin dosage (1 µg/mL) used to upregulate cPLA_2_-α expression was 10–100 higher than leptin levels in human circulation [42]. Cytokines such as IGF-1 and IL-6 are found to sensitize cells to leptin effects [43]. Leptin at 4–40 ng/mL enhances DU145 and PC-3 cells proliferation in the presence of 10% fetal bovine serum [44]. Thus, the presence of serum protein or cytokines may upregulate leptin effects. In this study, to known the pure signal transduction effect of leptin on cPLA_2_-α expression, A549 cells were treated with high concentrations (1 µg/mL) and long-term stimulation (24–48 h) of leptin in the absence of serum.

MAPKs, including p42/p44 MAPK, p38 MAPK, and JNK1/2, contribute to the occurrence or exacerbation of inflammation-related pathology [45]. It has been determined that inhibition of the c-Src/p38 MAPK pathway may ameliorate renal tubular epithelial cell apoptosis in diabetic mice [46]. Inhibition of the MAPK/NF-κB pathways by L2H17, a synthesized chalcone, also improved obesity-induced renal injury [47]. Both p42/p44 MAPK and JNK were determined to be involved in interferon-x03B3-induced hepatocyte inflammation [48]. Here, we conclude that leptin stimulated the phosphorylation of p42/p44 MAPK, p38 MAPK, and JNK1/2, separately. Pretreatment with U0126, PD98059 (inhibitors of MEK1/2), SB202190 (inhibitor of p38 MAPK), or SP600125 (inhibitor of JNK1/2) significantly reversed leptin induced mRNA and protein expression of cPLA_2_-α in A549 cells. These results indicate that leptin may affect lung inflammation through the activation of MAPKs pathways. This is similar to the effects of leptin on human bone marrow stromal cells and rat hepatocellular carcinoma, wherein p42/p44 MAPK or p38 MAPK play roles in modulating leptin signaling [49,50].

DNA is bound tightly to histone, but the binding loosens once genes are activated. The transcription activator p300 acetylates lysine residues on histone tails and reduces the interaction between histone and DNA. The activity of p300 is precisely regulated by various signaling components. Furthermore, p65 (also named RelA) is a component of NF-κB and participates in different biological processes, such as inflammation, cell growth, survival, immune response, and development [51]. Phosphorylation of the well-conserved p65 (Ser276) is a prerequisite and is considered a decisive factor for the p65:CBP/p300 interaction [52]. Studies have determined that transcriptional associations with HIF-1α, NF-κB, and β-catenin/p300 complexes contribute to hypoxic condition-changed tumor cell kinetics in endometrial carcinomas [53]. By regulating CD59 protein, NF-κB and CBP/p300 proteins protect host cells from complement attack [54]. We determined that leptin triggered the Ser276 phosphorylation of p65 in a time-dependent manner; however, this phosphorylation was attenuated by pretreatment with the OB-R antibody, U0126, SP600125, and Bay11-7082. These results revealed that leptin increased p65 phosphorylation through OB-R-dependent activation of the p42/p44 MAPK and JNK1/2 pathways. Moreover, leptin alone stimulated the activation of p300, which resulted in acetylation of histone H4. p300 phosphorylation and histone H4 acetylation were reversed by the OB-R antibody, U0126, SP600125, and Bay11-7082, suggesting the participation of OB-R, p42/p44 MAPK, JNK1/2, and NF-κB in leptin effects. We also found that pretreatment with SB202190 did not inhibit leptin-regulated p65 phosphorylation but attenuated p300 phosphorylation. This indicated that p38 MAPK may not contribute to p300 phosphorylation by enhancing NF-κB activity. The detailed effects of p38 MAPK on leptin-regulated p300 activity must be examined in the future. Bay11-7082 (an inhibitor of NF-κB), garcinol (an inhibitor of p300), and p300 siRNA all abolished leptin-stimulated cPLA_2_-α gene expression. This suggests that leptin mediates cPLA_2_-α expression, at least through OB-R-dependent activation of p42/p44 MAPK, JNK1/2, NF-κB, and the p300 cascade.

Based on the literature and our findings, we conclude that leptin stimulates mRNA and protein expression of cPLA_2_-α both *in vitro* in lung epithelial type II A549 cells and *in vivo* in ICR mice. Activation of p42/p44 MAPK, JNK1/2, NF-κB and p300 were necessary for the expression of cPLA_2_-α. OB-R-dependent activation of MAPKs and NF-κB regulated the phosphorylation of p300 and acetylation of histone H4. Finally, the transcription of *cPLA_2_*-α genes was enhanced through the activated OB-R/MAPKs/NF-κB/p300 cascade. The mechanisms of leptin-stimulated cPLA_2_-α expression may be a link between obesity and lung inflammation-related pathologies, suggesting novel strategies for treatment.

## 4. Experimental Section

### 4.1. Materials

Fetal bovine serum (FBS), DMEM/F-12 medium, and TRIZOL were purchased from Invitrogen (Carlsbad, CA, USA). Antibodies against cPLA_2_-α, OB-R, phosphor-p65, p300, phospho-p300, acetyl-Histone H4, p300 siRNA, and scrambled siRNA were obtained from Santa Cruz Biotechnology (Santa Cruz, CA, USA). PhosphoPlus p42/p44 MAPK, PhosphoPlusp38 MAPK, and phospho-JNK1/2 antibody kits were obtained from New England Biolabs (Beverly, MA, USA). The anti-GAPDH antibody was obtained from Novus Biologicals (Littleton, CO, USA). PD98059, U0126, SB202190, SP600125, and garcinol were obtained from Biomol (Plymouth Meeting, PA, USA). Leptin was obtained from BioVision (Milpitas, CA, USA). Hybond C membrane and Hyperfilms were obtained from GE Healthcare Biosciences (Buckinghamshire, UK). An enhanced chemiluminescence (ECL) Western blotting detection system was obtained from Visual Protein Biotechnology Co. (Taipei, Taiwan). The enzymes and other chemicals were obtained from Sigma (St. Louis, MO, USA).

### 4.2. Cell Culture of Human Alveolar Epithelial Cell Carcinoma (A549)

A549 cells (human alveolar epithelial cell carcinoma) were cultured as previously described [55]. After confluence was reached, the cells were treated with 0.05% trypsin/0.53 mM EDTA for 5 min at 37 °C. The cell suspension was diluted with DMEM/F-12 containing a 10% concentration FBS to reach a cell concentration of 2 × 10^5^ cells/mL. The cell suspension was plated in 12-well culture plates (1 mL per well) or (10 mL per dish) 10-cm culture dishes for the measurement of protein expression and mRNA accumulation.

### 4.3. Animal Treatment

Male ICR mice aged 4 wks were purchased from the BioLASCO Taiwan Co., Ltd. (Taipei, Taiwan) and handled according to the guidelines of the Animal Care Committee of Fu Jen Catholic University and the NIH Guide for the Care and Use of Laboratory Animals. ICR mice were anesthetized with pentobarbital (60 mg/kg) *i.p.* and placed individually on a board in a near vertical position [56]. Their tongues were withdrawn with lined forceps. Leptin (2 mg/kg) was placed posterior in the throat and aspirated into the lungs. Control mice were administrated sterile 0.1% BSA. Mice awoke unassisted after 10–20 min. After 4, 24, or 48 h of leptin treatments, the mice were sacrificed and lung tissues were extracted for protein and mRNA expression of cPLA_2_-α, GAPDH, or β-actin analysis.

### 4.4. Isolation of BAL

After 4, 24, or 48 h of leptin treatment, the mice were anesthetized and BAL was performed through a tracheal cannula by using 1-mL aliquots of ice-cold PBS medium [56]. BAL samples were centrifuged at 500× *g* at 4 °C, and cell pellets were washed and resuspended in PBS. Cell counts were determined using the scil Vet ABC™ Hematology Analyzer (scil animal care company, Inc., Gurnee, IL, USA).

### 4.5. Transfection with Small Interference RNA

A549 cells were plated in 3 × 10^5^ cells/mL (1 mL/well) in 12-well culture plates for 24 h, reaching approximately 80% confluence [29]. The cells were replaced with 0.4 mL of DMEM/F-12 containing 10% FBS. The DNA Metafectene reagent complex was prepared according to manufacturer instructions (Biontex, Martinsried, Planegg, Germany). The amount of transfected DNA was maintained constant with 100 nM scrambled or p300 siRNA for each well. The DNA METAFECTENE complex (0.1 mL) was added to each well and then incubated at 37 °C for 24 h. After 24 h of transfection, the cells were washed twice with PBS and maintained in DMEM/F-12 medium for 72 h (before treatment with leptin for the indicated time intervals).

### 4.6. Protein Extraction and Western Blot

After leptin stimulation, the cells were rapidly washed with ice-cold PBS, scraped, and collected through centrifugation at 1000× *g* for 10 min [56]. The collected cells were lysed with an ice-cold lysis buffer. The lysates were centrifuged at 45,000× *g* for 1 h at 4 °C to yield the whole cell extract. Samples from these supernatant fractions (30 µg protein) were subjected to SDS-PAGE by using a 10% or 12% concentration running gel. Proteins were transferred to nitrocellulose membranes, which were then successively incubated at room temperature with 5% BSA in TTBS for 1 h. The membranes were incubated overnight at 4 °C with an anti-cPLA_2_, anti-OB-R, antiphospho-p42/p44 MAPK, antiphospho-p38 MAPK, antiphospho-JNK1/2, antiphospho-p65, anti-p300, antiphospho-p300, antiacetyl histone H4, or the anti-GAPDH antibody according to manufacturer recommendations. The membranes were incubated with a 1:2000 dilution of an anti-mouse or anti-rabbit horseradish peroxidase antibody for 1 h. The immunoreactive bands detected using ECL reagents were developed by Hyperfilm-ECL.

### 4.7. Total RNA Extraction and Gene Expression Analysis

Total RNA was extracted from A549 cells by using Trizol, as previously described [55]. The cDNA containing 2 µg of RNA was used as a template to analyze cPLA_2_-α mRNA levels. Oligonucleotide primers for β-actin, cPLA_2_-α, and OB-R were as follows: for β-actin: 5′-TGACGGGGTCACCCACACTGTGCCCATCTA-3′ (sense), 5′-CTAGAAGCATTTGCGGTGGACGATG-3′ (antisense); for cPLA_2_-α: 5′-CTCACACCACAGAAAGTTAAAAGAT-3′ (sense), 5′-GCTACCACAGGCACATCACG-3′ (antisense); for common OB-R: 5′-ATCCCCATTGAGAAGTACCAG-3′ (sense); for OB-R (short-form): 5’-GAAGTTGGCACATTGGGTTC-3’ (antisense); for OB-R (medium-form): 5’-AATAGTGGAGGGAGGGTCAG-3’ (antisense) and for OB-R (long-form) 5’-TGTCCTGGAGAACTCTGATC-3’ (antisense). The amplification profile included one cycle of initial denaturation at 94 °C for 5 min; 30 cycles of denaturation at 94 °C for 1 min; primer annealing at 58 °C (cPLA_2_-α), 56 °C (OB-R), and 60 °C (β-actin) for 1 min; extension at 72 °C for 1 min; and one cycle of final extension at 72 °C for 5 min. The expression of β-actin was used as an internal control for the assay of a constitutively expressed gene.

### 4.8. Statistical Analysis of Data

All data are expressed as the mean ± standard error of the mean by using the GraphPad Prism Program (GraphPad, San Diego, CA, USA) [12]. Quantitative data were analyzed using one-way ANOVA followed by Tukey’s post hoc test at a *p* < 0.05 level of significance. All of the experiments were performed at least 5 times.

## References

[1] McClean K.M., Kee F., Young I.S., Elborn J.S. (2008). Obesity and the lung: 1. Epidemiology. Thorax.

[2] Franssen F.M., O’Donnell D.E., Goossens G.H., Blaak E.E., Schols A.M. (2008). Obesity and the lung: 5. Obesity and COPD. Thorax.

[3] Sin D.D., Sutherland E.R. (2008). Obesity and the lung: 4. Obesity and asthma. Thorax.

[4] Crummy F., Piper A.J., Naughton M.T. (2008). Obesity and the lung: 2. Obesity and sleep-disordered breathing. Thorax.

[5] Konter J., Baez E., Summer R.S. (2013). Obesity: “Priming” the lung for injury. Pulm. Pharmacol. Ther..

[6] Beuther D.A., Sutherland E.R. (2007). Overweight, obesity, and incident asthma: A meta-analysis of prospective epidemiologic studies. Am. J. Respir. Crit. Care Med..

[7] Grunstein R.R., Stenlof K., Hedner J.A., Peltonen M., Karason K., Sjostrom L. (2007). Two year reduction in sleep apnea symptoms and associated diabetes incidence after weight loss in severe obesity. Sleep.

[8] Lee I.T., Lee C.W., Tung W.H., Wang S.W., Lin C.C., Shu J.C., Yang C.M. (2010). Cooperation of TLR2 with MyD88, PI3K, and Rac1 in lipoteichoic acid-induced cPLA2/COX-2-dependent airway inflammatory responses. Am. J. Pathol..

[9] Luo S.F., Lin W.N., Yang C.M., Lee C.W., Liao C.H., Leu Y.L., Hsiao L.D. (2006). Induction of cytosolic phospholipase a2 by lipopolysaccharide in canine tracheal smooth muscle cells: Involvement of MAPKs and NF-κB pathways. Cell Signal..

[10] Guillemot L., Medina M., Pernet E., Leduc D., Chignard M., Touqui L., Wu Y. (2014). Cytosolic phospholipase A2α enhances mouse mortality induced by pseudomonas aeruginosa pulmonary infection via interleukin 6. Biochimie.

[11] Nagase T., Uozumi N., Ishii S., Kume K., Izumi T., Ouchi Y., Shimizu T. (2000). Acute lung injury by sepsis and acid aspiration: A key role for cytosolic phospholipase A2. Nat. Immunol..

[12] Lin W.N., Lin C.C., Cheng H.Y., Yang C.M. (2011). Regulation of cyclooxygenase-2 and cytosolic phospholipase *A2* gene expression by lipopolysaccharide through the RNA-binding protein HuR: Involvement of NADPH oxidase, reactive oxygen species and mitogen-activated protein kinases. Br. J. Pharmacol..

[13] Otto T.C., Lane M.D. (2005). Adipose development: From stem cell to adipocyte. Crit. Rev. Biochem. Mol. Biol..

[14] Galic S., Oakhill J.S., Steinberg G.R. (2010). Adipose tissue as an endocrine organ. Mol. Cell. Endocrinol..

[15] Zhang F., Basinski M.B., Beals J.M., Briggs S.L., Churgay L.M., Clawson D.K., DiMarchi R.D., Furman T.C., Hale J.E., Hsiung H.M. (1997). Crystal structure of the obese protein leptin-e100. Nature.

[16] La Cava A., Matarese G. (2004). The weight of leptin in immunity. Nat. Rev. Immunol..

[17] De Rosa V., Procaccini C., Cali G., Pirozzi G., Fontana S., Zappacosta S., la Cava A., Matarese G. (2007). A key role of leptin in the control of regulatory T cell proliferation. Immunity.

[18] Watowich S.S., Wu H., Socolovsky M., Klingmuller U., Constantinescu S.N., Lodish H.F. (1996). Cytokine receptor signal transduction and the control of hematopoietic cell development. Annu. Rev. Cell Dev. Biol..

[19] Houseknecht K.L., Baile C.A., Matteri R.L., Spurlock M.E. (1998). The biology of leptin: A review. J. Anim. Sci..

[20] Wang M.Y., Zhou Y.T., Newgard C.B., Unger R.H. (1996). A novel leptin receptor isoform in rat. FEBS Lett..

[21] Stofkova A. (2009). Leptin and adiponectin: From energy and metabolic dysbalance to inflammation and autoimmunity. Endocr. Regul..

[22] Gong Y., Ishida-Takahashi R., Villanueva E.C., Fingar D.C., Munzberg H., Myers M.G. (2007). The long form of the leptin receptor regulates STAT5 and ribosomal protein S6 via alternate mechanisms. J. Biol. Chem..

[23] Banks A.S., Davis S.M., Bates S.H., Myers M.G. (2000). Activation of downstream signals by the long form of the leptin receptor. J. Biol. Chem..

[24] Bjorbaek C., Buchholz R.M., Davis S.M., Bates S.H., Pierroz D.D., Gu H., Neel B.G., Myers M.G., Flier J.S. (2001). Divergent roles of SHP-2 in ERK activation by leptin receptors. J. Biol. Chem..

[25] Miao D., Zhang L. (2015). Leptin modulates the expression of catabolic genes in rat nucleus pulposus cells through the mitogenactivated protein kinase and janus kinase 2/signal transducer and activator of transcription 3 pathways. Mol. Med. Rep..

[26] Cheng S.E., Lin C.C., Lee I.T., Hsu C.K., Kou Y.R., Yang C.M. (2011). Cigarette smoke extract regulates cytosolic phospholipase A2 expression via NADPH oxidase/MAPKs/AP-1 and p300 in human tracheal smooth muscle cells. J. Cell. Biochem..

[27] Chi P.L., Luo S.F., Hsieh H.L., Lee I.T., Hsiao L.D., Chen Y.L., Yang C.M. (2011). Cytosolic phospholipase A2 induction and prostaglandin E2 release by interleukin-1β via the myeloid differentiation factor 88-dependent pathway and cooperation of p300, AKT, and NF-βB activity in human rheumatoid arthritis synovial fibroblasts. Arthritis Rheum..

[28] Lee C.W., Lin W.N., Lin C.C., Luo S.F., Wang J.S., Pouyssegur J., Yang C.M. (2006). Transcriptional regulation of VCAM-1 expression by tumor necrosis factor-alpha in human tracheal smooth muscle cells: Involvement of MAPKs, NF-κB, p300, and histone acetylation. J. Cell. Physiol..

[29] Lin W.N., Luo S.F., Lee C.W., Wang C.C., Wang J.S., Yang C.M. (2007). Involvement of MAPKs and NF-κB in LPS-induced VCAM-1 expression in human tracheal smooth muscle cells. Cell Signal..

[30] Wang C.C., Lin W.N., Lee C.W., Lin C.C., Luo S.F., Wang J.S., Yang C.M. (2005). Involvement of p42/p44 mapk, p38 mapk, jnk, and nf-kappab in il-1beta-induced VCAM-1 expression in human tracheal smooth muscle cells. Am. J. Physiol. Lung Cell. Mol. Physiol..

[31] Chi P.L., Chen Y.W., Hsiao L.D., Chen Y.L., Yang C.M. (2012). Heme oxygenase 1 attenuates interleukin-1β-induced cytosolic phospholipase A2 expression via a decrease in NADPH oxidase/reactive oxygen species/activator protein 1 activation in rheumatoid arthritis synovial fibroblasts. Arthritis Rheum..

[32] Li H., Wang Y.P., Zhang L.N., Tian G. (2014). Perivascular adipose tissue-derived leptin promotes vascular smooth muscle cell phenotypic switching via p38 mitogen-activated protein kinase in metabolic syndrome rats. Exp. Biol. Med..

[33] Li Z., Yu X., Liang J., Wu W.K., Yu J., Shen J. (2014). Leptin downregulates aggrecan through the p38-adamst pathway in human nucleus pulposus cells. PLoS ONE.

[34] Shalini V., Jayalekshmi A., Helen A. (2015). Mechanism of anti-inflammatory effect of tricin, a flavonoid isolated from Njavara rice bran in LPS induced hPBMCs and carrageenan induced rats. Mol. Immunol..

[35] Dattaroy D., Pourhoseini S., Das S., Alhasson F., Seth R.K., Nagarkatti M., Michelotti G.A., Diehl A.M., Chatterjee S. (2015). Micro-RNA 21 inhibition of SMAD7 enhances fibrogenesis via leptin-mediated NADPH oxidase in experimental and human nonalcoholic steatohepatitis. Am. J. Physiol. Gastrointest. Liver Physiol..

[36] Huertas A., Tu L., Thuillet R., le Hiress M., Phan C., Ricard N., Nadaud S., Fadel E., Humbert M., Guignabert C. (2015). Leptin signalling system as a target for pulmonary arterial hypertension therapy. Eur. Respir. J..

[37] Oh Y.M., Jeong B.H., Woo S.Y., Kim S.Y., Kim H., Lee J.H., Lim S.Y., Rhee C.K., Yoo K.H., Lee J.H. (2015). Association of plasma adipokines with chronic obstructive pulmonary disease severity and progression. Ann. Am. Thorac. Soc..

[38] Mendivil C.O., Koziel H., Brain J.D. (2015). Metabolic hormones, apolipoproteins, adipokines, and cytokines in the alveolar lining fluid of healthy adults: Compartmentalization and physiological correlates. PLoS ONE.

[39] Ubags N.D., Vernooy J.H., Burg E., Hayes C., Bement J., Dilli E., Zabeau L., Abraham E., Poch K.R., Nick J.A. (2014). The role of leptin in the development of pulmonary neutrophilia in infection and acute lung injury. Crit. Care Med..

[40] Mancuso P., Canetti C., Gottschalk A., Tithof P.K., Peters-Golden M. (2004). Leptin augments alveolar macrophage leukotriene synthesis by increasing phospholipase activity and enhancing group IVC iPLA2 (cPLA2γ) protein expression. Am. J. Physiol. Lung Cell. Mol. Physiol..

[41] Suzukawa M., Koketsu R., Baba S., Igarashi S., Nagase H., Yamaguchi M., Matsutani N., Kawamura M., Shoji S., Hebisawa A. (2015). Leptin enhances ICAM-1 expression, induces migration and cytokine synthesis, and prolongs survival of human airway epithelial cells. Am. J. Physiol. Lung Cell. Mol. Physiol..

[42] Miyazaki T., Bub J.D., Iwamoto Y. (2008). c-Jun NH 2-terminal kinase mediates leptin-stimulated androgen-independent prostate cancer cell proliferation via signal transducer and activator of transcription 3 and Akt. Biochim. Biophys. Acta.

[43] Onuma M., Bub J.D., Rummel T.L., Iwamoto Y. (2003). Prostate cancer cell-adipocyte interaction: Leptin mediates androgen-independent prostate cancer cell proliferation through c-jun NH 2-terminal kinase. J. Biol. Chem..

[44] Somasundar P., Frankenberry K.A., Skinner H., Vedula G., McFadden D.W., Riggs D., Jackson B., Vangilder R., Hileman S.M., Vona-Davis L.C. (2004). Prostate cancer cell proliferation is influenced by leptin. J. Surg. Res..

[45] Cheng C.Y., Hsieh H.L., Sun C.C., Lin C.C., Luo S.F., Yang C.M. (2009). Il-1 β induces urokinase-plasminogen activator expression and cell migration through PKC α, JNK1/2, and NF-κB in A549 cells. J. Cell. Physiol..

[46] Haijiang W., Yonghong S., Xinna D., Ye S., Chunyang D., Jinying W., Yunzhuo R., Ming W., Yanjuan H., Huijun D. (2015). Inhibition of c-Src/p38 MAPK pathway ameliorates renal tubular epithelial cells apoptosis in db/db mice. Mol. Cell. Endocrinol..

[47] Fang Q., Deng L., Wang L., Zhang Y., Weng Q., Yin H., Pan Y., Tong C., Wang J., Liang G. (2015). Inhibition of MAPKs/NF-κB-dependent inflammation by a novel chalcone protects kidney from high fat diet-induced injuries in mice. J. Pharmacol. Exp. Ther..

[48] Wu S., Xue J., Yang Y., Zhu H., Chen F., Wang J., Lou G., Liu Y., Shi Y., Yu Y. (2015). Isoliquiritigenin inhibits interferon-γ-inducible genes expression in hepatocytes through down-regulating activation of JAK1/STAT1, IRF3/MyD88, ERK/MAPK, JNK/MAPK and PI3K/Akt signaling pathways. Cell. Physiol. Biochem..

[49] Kim G.S., Hong J.S., Kim S.W., Koh J.M., An C.S., Choi J.Y., Cheng S.L. (2003). Leptin induces apoptosis via ERK/cPLA2/cytochrome c pathway in human bone marrow stromal cells. J. Biol. Chem..

[50] Thompson K.J., Lau K.N., Johnson S., Martinie J.B., Iannitti D.A., McKillop I.H., Sindram D. (2011). Leptin inhibits hepatocellular carcinoma proliferation via p38-MAPK-dependent signalling. HPB.

[51] Sun S.C. (2011). Non-canonical NF-κB signaling pathway. Cell Res..

[52] Mukherjee S.P., Behar M., Birnbaum H.A., Hoffmann A., Wright P.E., Ghosh G. (2013). Analysis of the RelA: CBP/p300 interaction reveals its involvement in NF-κB-driven transcription. PLoS Biol..

[53] Yoshida T., Hashimura M., Mastumoto T., Tazo Y., Inoue H., Kuwata T., Saegusa M. (2013). Transcriptional upregulation of HIF-1α by NF-κB/p65 and its associations with β-catenin/p300 complexes in endometrial carcinoma cells. Lab. Investig..

[54] Chung M.H., Kim D.H., Na H.K., Kim J.H., Kim H.N., Haegeman G., Surh Y.J. (2014). Genistein inhibits phorbol ester-induced NF-κB transcriptional activity and COX-2 expression by blocking the phosphorylation of p65/RelA in human mammary epithelial cells. Mutat. Res..

[55] Lee I.T., Lin C.C., Lin W.N., Wu W.L., Hsiao L.D., Yang C.M. (2013). Lung inflammation caused by adenosine-5′-triphosphate is mediated via Ca^2+^/PKCs-dependent COX-2/PGE2 induction. Int. J. Biochem. Cell Biol..

[56] Lin W.N., Luo S.F., Lin C.C., Hsiao L.D., Yang C.M. (2009). Differential involvement of PKC-dependent MAPKs activation in lipopolysaccharide-induced AP-1 expression in human tracheal smooth muscle cells. Cell Signal..

